# Retraction: Extracellular vesicles long non-coding RNA AGAP2-AS1 contributes to cervical cancer cell proliferation through regulating the miR-3064-5p/SIRT1 axis

**DOI:** 10.3389/fonc.2023.1145873

**Published:** 2023-01-31

**Authors:** Frontiers Editorial Office

**Keywords:** cervical cancer, proliferation, lncRNA AGAP2-AS1, miR-3064-5p, Sirtuin 1 (SIRT1)

Please find the retraction statement below:

“Following publication, concerns were raised regarding the integrity of the images in the published figures. The authors failed to provide a satisfactory explanation during the investigation, which was conducted in accordance with Frontiers’ policies.

This retraction was approved by the Chief Editors of Frontiers in Oncology and the Chief Executive Editor of Frontiers.”

